# Toward Improved Lifetimes of Organic Solar Cells under Thermal Stress: Substrate-Dependent Morphological Stability of PCDTBT:PCBM Films and Devices

**DOI:** 10.1038/srep15149

**Published:** 2015-10-15

**Authors:** Zhe Li, Kar Ho Chiu, Raja Shahid Ashraf, Sarah Fearn, Rajeev Dattani, Him Cheng Wong, Ching-Hong Tan, Jiaying Wu, João T. Cabral, James R. Durrant

**Affiliations:** 1 Department of Chemistry, Centre for Plastic Electronics, Imperial College London, London SW7 2AZ; 2 Department of Chemical Engineering, Centre for Plastic Electronics, Imperial College London, London SW7 2AZ; 3 Department of Materials, Centre for Plastic Electronics, Imperial College London, London SW7 2AZ; 4 SPECIFIC, College of Engineering, Swansea University, Baglan Bay Innovation, Knowledge Centre, Central Avenue, Baglan SA12 7AX; 5 Engineering Product Development, Singapore University of Technology and Design, 8 Somapah Road, 487372 Singapore

## Abstract

Morphological stability is a key requirement for outdoor operation of organic solar cells. We demonstrate that morphological stability and lifetime of polymer/fullerene based solar cells under thermal stress depend strongly on the substrate interface on which the active layer is deposited. In particular, we find that the stability of benchmark PCDTBT/PCBM solar cells under modest thermal stress is substantially increased in inverted solar cells employing a ZnO substrate compared to conventional devices employing a PEDOT:PSS substrate. This improved stability is observed to correlate with PCBM nucleation at the 50 nm scale, which is shown to be strongly influenced by different substrate interfaces. Employing this approach, we demonstrate remarkable thermal stability for inverted PCDTBT:PC_70_BM devices on ZnO substrates, with negligible (<2%) loss of power conversion efficiency over 160 h under 85 °C thermal stress and minimal thermally induced “burn-in” effect. We thus conclude that inverted organic solar cells, in addition to showing improved environmental stability against ambient humidity exposure as widely reported previously, can also demonstrate enhanced morphological stability. As such we show that the choice of suitable substrate interfaces may be a key factor in achieving prolonged lifetimes for organic solar cells under thermal stress conditions.

Organic photovoltaic (OPV) solar cells attract significant interest due to their potential in ease of processing, low cost and high flexibility. Over the past decade, steady increases in power conversion efficiency (PCE) have resulted in record 9–10% PCE owing to improved material and device design, a threshold level considered necessary for commercial viability^1^,^2^,^3^,^4^. However, achieving operating lifetimes long enough for commercial application remains a major challenge. A number of environmental factors including heat, light, humidity and oxygen exposure are known to cause rapid degradation of their performance during operation^5^. To ensure long durability of OPV devices, the cause and mechanism of degradation under each of these stress factors needs to be identified and addressed. In this paper, we focus on device stability under thermal stress, tackling the role of the bottom electrode in the morphological stability of the device photoactive layer.

Organic bulk heterojunction solar cells are typically based on blend films containing conjugated polymers and soluble fullerene derivatives such as PC_60_BM and PC_70_BM. Achieving an optimised blend morphology at the nanometer scale is crucial to their efficient operation. Furthermore, ensuring that such blend morphology is stable over time, particularly under the thermal stresses relevant to operating conditions, remains a key challenge for outdoor application of OPV devices. Recently the diffusion of PCBM throughout the blend film, as a small molecule with low intermolecular interactions, has been proposed as a primary cause of thermally induced degradation of OPV devices^6^,^7^. Under thermal annealing conditions, in particular above the glass transition temperature (T_g_) of the blend, OPV morphologies are generally found to coarsen with time and PCBM nucleates and grows to form aggregates or crystallites on length scales from 10 s of nm to microns, with the number density, size and shape of these crystallites depending upon the choice of polymer:fullerene blend, processing conditions and thermal annealing conditions^8^,^9^. For instance micron-sized PCBM needles generally grow within P3HT and PS thin films on SiO_x_^9^, or chromosome/sheaf-like crystals within PCDTBT^8^,^9^,^10^,^11^, and only nano-crystals within PCDTBT onto PEDOT:PSS substrates^9^,^12^. Such crystallisation at polymer interfaces has been found to be bimodal, yielding a morphology dominated by either nano- or micro-sized crystals^9^,^10^,^13^,^14^,^15^, and mediated by the polymer phase^16^, depending on substrate, annealing temperature and polymer matrix. Formation of these PCBM crystallites has been correlated with loss of OPV device performance, thought to arise from poor charge generation/transport, as well as mechanical problems such as electrode delamination^7^,^8^. To address the issue of PCBM diffusion/crystallisation and to improve the morphological stability of OPV devices, a number of approaches have been developed including the use of high T_g_ materials^17^,^18^, chemical crosslinking of one or both of the donor/acceptor domains^19^,^20^, light induced PCBM oligomerisation^9^,^10^,^11^ and the addition of fullerene dimers^12^. However, achieving morphological stability of OPV devices on the timescales required for commercial application without compromising material complexity (and cost) and/or device efficiency remains a significant challenge.

The insertion of an interfacial buffer layer in between the device TCO electrode and the photoactive layer is commonly used to improve the efficiency of OPV devices^4^,^21^,^22^. Depending on their workfunctions, a broad variety of interlayer materials such as TiO_x_, ZnO, PEDOT:PSS, Ca, and MoO_3_ have been used either as electron transporting layers (ETL) or hole transporting layers (HTL). The use of interlayers typically results in enhanced fill factors (FF) and current densities of OPV devices by enabling, for example, selective carrier extraction. More detrimentally, a range of interlayers such as Ca and PEDOT:PSS are known to lead to severe degradation of unsealed devices in ambient environment, due to diffusion and subsequent chemical reaction of oxygen and water with the interlayer materials. Most research effort on device interlayers to date has been focused either on the efficiency improvement or chemical degradation of OPV devices under environmental stress conditions of water and oxygen exposure, with studies under thermal stress being limited to analyses of film morphological behaviour, without direct correlation with device stability measurements^5^. In particular it has been shown that the use of inverted device architectures, where the bottom PEDOT:PSS interlayer is replaced by an oxide ETL such as ZnO, can result in improved device resistance to humidity induced degradation^5^. However the impact of such inverted device architectures upon morphological and device stability under thermal stress has not received significant attention to date.

Thermally induced degradation of OPV devices has been described as a two-stage process, with an initial PCE loss of 10–20% within the first 100s of minutes (also described as a thermal “burn-in” process^18^) followed by a similar loss of PCE in the next 100s to 1000s of hours^9^,^23^. (We note these two ‘stages’ may result from a single underlying process.) As the thermal “burn-in” process typically accounts for a significant proportion of device degradation within a short period, understanding and eliminating PCE losses caused by thermal “burn-in” is essential to achieving long term operational stability of OPV devices. Nevertheless, very limited research efforts have so far been dedicated to this topic and the origin of the thermal “burn-in” remains unclear. Sachs-Quintana *et al.*^18^ reported the formation of a thin, polymer rich layer acting as an electron barrier at the top electrode/active layer interface at annealing temperatures above the polymer T_g_ for P3HT:PCBM blends. The authors also found that this barrier can be removed by replacing the top electrode of thermally aged devices and thus the device performance can be restored, suggesting an interfacial origin of the thermal “burn-in” process. In this paper we provide complementary insight into this process, linking the thermally induced degradation of fullerene based solar cells (especially the thermal “burn-in” effect) to the formation of nm-sized PCBM crystallites under modest thermal stress relevant to typical operating conditions (at temperatures far below the polymer T_g_). Through a side-by-side comparison of the morphological and device behaviour of the benchmark PCDTBT:PCBM blend system on two substrate types that are extensively used for OPV device fabrication, PEDOT:PSS and ZnO, we show that the thermal “burn-in” process is strongly dependent upon the bottom interface on which the active layer is deposited. We further demonstrate superior thermal stability of devices based on ZnO substrates (Supplementary Fig. S1) which were found to show significantly suppressed nm-scale PCBM crystallisation compared to PEDOT:PSS, thereby providing an attractive approach to control the blend morphology and improve the operating stability of OPV devices by varying their interfacial properties.

## Results and Discussion

Our study is focused on investigating the influence of substrates on the morphological behaviour of OPV blend films and devices under thermal annealing conditions. (See Supplementary Fig. S2 for an illustration of our methodology). A blend of the benchmark carbazole donor polymer PCDTBT with PCBM was selected as a model OPV system and three ubiquitous, representative and well characterised substrate types, namely SiO_x_, ZnO and PEDOT:PSS were chosen for morphological stability studies.

**Figure 1:**
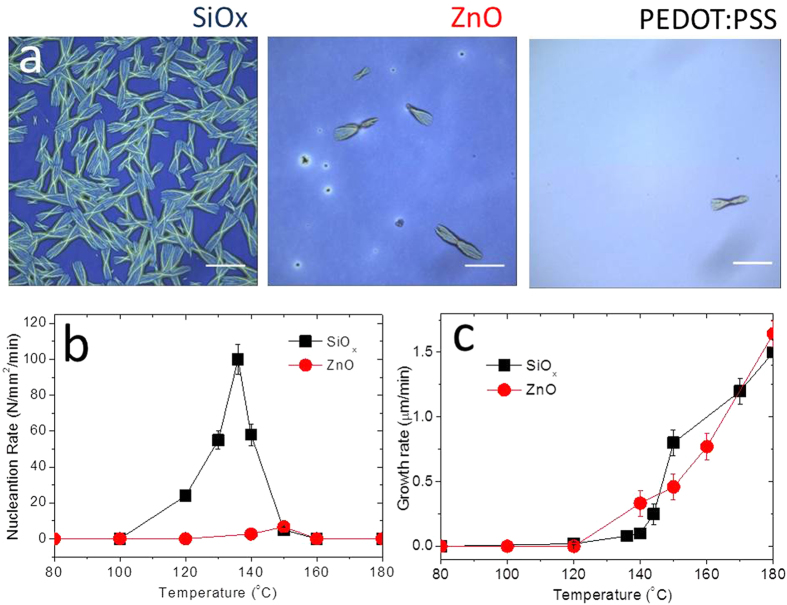
Optical microscopy and micron-scale nucleation/growth analysis of PCDTBT:PC_60_BM blend films on different substrate types. (**a**) Optical micrographs of PCDTBT:PC_60_BM (1:2) thin film mixtures, 85–100 nm thick, annealed at 140 °C for 1 h in N_2_ environment, supported by three different substrates: SiO_x_, ZnO and PEDOT:PSS (Scale bar: 25 μm). (**b**) Temperature dependence of the micron-scale nucleation rate, obtained after 1 h annealing, for SiO_x_ and ZnO substrates. (**c**) Temperature dependence of the corresponding crystallite growth rate. (PC_60_BM crystallisation data on SiO_x_ from our previous study [9]). Nucleation and growth rates were estimated by the initial change of crystal number density and length for annealing times up to 60 minutes, at each annealing temperature. Uncertainties were estimated by the maximum deviation obtained from 10 separate measurements, with a scan area of 0.025 mm^2^.

We start by building upon previous reports that strong thermal annealing of PCDTBT:PCBM blend films above a threshold temperature results in the formation of micron-sized PCBM crystallites on SiO_x_ substrates visible under an optical microscope^8^,^10^,^11^. Figure 1(a) shows optical micrographs of PCDTBT:PC_60_BM blend films annealed at 140 °C for 1 h on different substrates. While all as cast films are uniform and free of macroscopic PC_60_BM crystallites, remarkable differences are observed upon thermally annealing films on the different substrate types. In particular, densely populated PC_60_BM crystallites form on SiO_x_ substrates, with a number density of ≈7000 mm^−2^ and average size of ≈30 μm (Fig. 1a, left). In comparison, thermally annealed films on ZnO (Fig. 1a, middle) and PEDOT:PSS substrates (Fig. 1a, right) show a significantly reduced number density of the macroscopic PC_60_BM crystallites (≈160 mm^−2^ for ZnO substrates and ≈0.01 mm^−2^ for PEDOT:PSS substrates), albeit with very similar shape (chromosome shaped) and size (≈30 μm) to those formed on SiO_x_ substrates. Qualitatively, we find that these observations correlate with the substrates surface energy (Supplementary Fig. S3), with the highest surface energy substrate SiO_x_ (≈70 mJ/m^2^) exhibiting the largest number of micron-sized crystallites, and PEDOT:PSS (≈40–60 mJ/m^2^) the fewest. Figure 1(b,c) show the nucleation and growth rates analysis of the thermally induced macroscopic PC_60_BM crystallites formation on SiO_x_ and ZnO substrates as a function of thermal annealing temperature (the number density of PC_60_BM crystallites on PEDOT:PSS substrates is too low to enable quantitative analysis and is thus not included in the rate comparison). Large differences in the temperature dependence of the nucleation rate are also found between the two substrates (approximately 20 °C difference in temperature of the max nucleation peak and nearly 10 times in absolute nucleation rate across the temperature range investigated). By contrast a monotonic increase of the growth rate is observed with increasing temperature, with similar growth rates for both SiO_x_ and ZnO substrates. The thermally-induced micron-scale PCBM crystallisation is evidently strongly dependent on substrate, mainly due to differences in the nucleation rate (which determines the number density of the thermally induced macroscopic PCBM crystallites), rather than the growth rate (size) or mechanism (defining shape). As such it is apparent that PCBM crystallisation under these conditions is primarily determined by the rate of crystal nucleation rather than PCBM diffusion kinetics.

**Figure 2:**
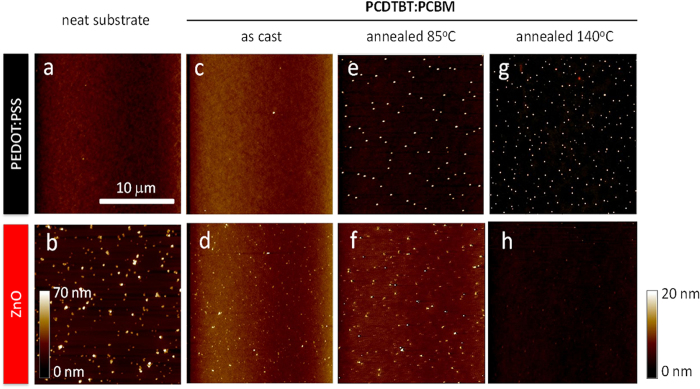
AFM height images of (**a**) neat PEDOT:PSS and (**b**) ZnO substrates; as cast PCDTBT:PC_60_BM (1:2) blend films (~85–100 nm thick) on (**c**) PEDOT:PSS and (**d**) ZnO substrates; blend films thermally annealed at 85 °C and 140 °C on (**e**,**g**) PEDOT:PSS and (**f**,**h**) ZnO substrates, respectively, investigating the nanoscale crystallisation behaviour of PC_60_BM. The height scale is Δz = 20 nm for all images, except (**b**) at 70 nm.

We next examine the nano-morphological behaviour of the blend films on different substrates with atomic force microscopy (AFM), under thermal stress conditions analogous to device operating conditions (see Supplementary Fig. S4 for representative line scans and rms roughness analysis of the AFM images). In particular we employ the 85 °C condition used as a PV industry standard^5^. We focus on the comparison of thermally induced PC_60_BM crystallisation on PEDOT:PSS and ZnO substrates. Indeed, no nano-scale crystallisation is found on SiO_x_, in agreement with the reported PCBM competitive bimodal crystallisation process^16^; further SiO_x_ is unsuitable for OPV device fabrication. Neat PEDOT:PSS substrates (Fig. 2a) and as cast blend films (Fig. 2c) are featureless and free of PC_60_BM crystallites. Given the rougher topography of ZnO surfaces, both the neat substrate (Fig. 2b) and as cast blend films (Fig. 2d) show characteristic nm-scale features, whose height is lower in the latter as partly covered by the blend film. Thermal annealing of the blend films at temperatures lower than the polymer T_g_ (measured as 106 ± 1 °C for PCDTBT) does not, as expected, lead to the formation of macroscopic PC_60_BM crystallites on either substrate type, attributed to a lack of long range mobility of the polymer and fullerene phases in the blend. However, as revealed by the AFM images, thermal annealing at 85 °C of blend films on PEDOT:PSS substrates leads to the formation of nm-sized features (Fig. 2e), confirmed to be PC_60_BM crystallites by TEM imaging (see Supplementary Fig. S5 for corresponding AFM images with arrows distinguishing nanoscale ZnO features and PCBM crystallites, and Supplementary Fig. S6 for TEM image confirming these nm-sized features to be PC_60_BM crystallites)^9^,^12^. The degree of crystallinity of smaller sub-micron PCBM aggregates cannot be determined from these experiments and, for simplicity, throughout this manuscript, both these, and micron-scale PCBM crystals, will be referred to as ‘crystallites’. We also note these are significantly larger than the smaller 3–10 nm PCBM aggregates often observed in optimised blends^15^. The appearance of these nm-sized features, with a dimension of ≈150 nm in diameter and ≈30 nm above the film surface, has been correlated with a degradation of device performance^9^,^12^. At a higher thermal annealing temperature of 140 °C, nm-sized PC_60_BM crystallites still form (Fig. 2g) but with a much higher number density (≈630000 mm^−2^) compared to samples annealed at 85 °C (≈220000 mm^−2^). By comparison, the blend films on ZnO substrates appear to become smoother under thermal annealing from 85 °C to 140 °C (Fig. 2f,h), attributed to increased planarisation of the underlying roughness of the ZnO layer by the photoactive blend. These observations indicate that thermally induced PCBM crystallisation on PEDOT:PSS and ZnO substrates are fundamentally different. ZnO substrates appear to be effective at suppressing both micron- and nano-scale PCBM crystallization mechanisms within the temperature range studied. PCDTBT:PCBM blend films cast on ZnO substrates have thus significantly higher morphological stability on a nm-scale (and μm, within this operationally relevant temperature range) compared to films cast on PEDOT:PSS substrates.

**Figure 3:**
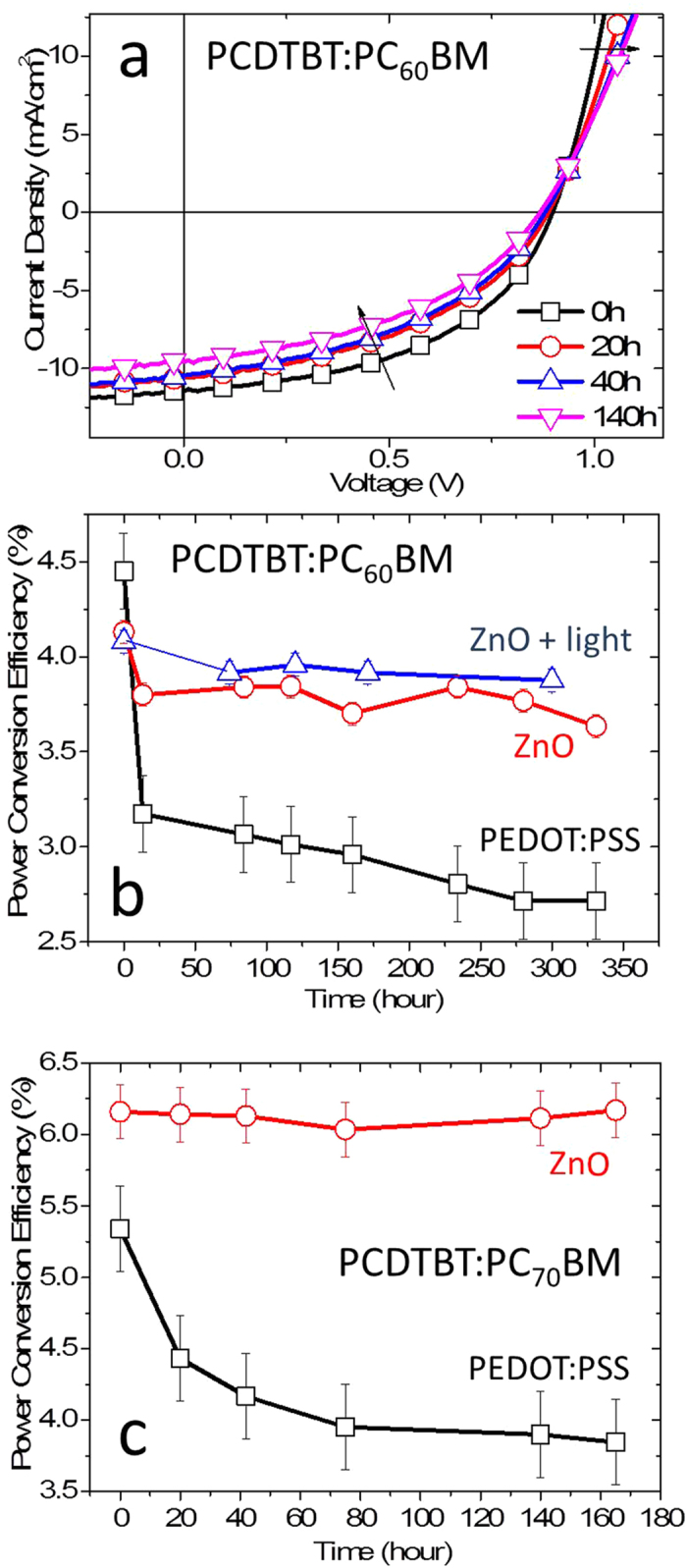
Device stability tests of PCDTBT:PC_60_BM and PCDTBT:PC_70_BM solar cells under modest thermal stress. (**a**) Time evolution of the J-V characteristics of a typical PCDTBT:PC_60_BM solar cell with a conventional ITO/PEDOT:PSS/PCDTBT:PC_60_BM/Ca/Al architecture under 85 °C thermal stress. (**b**) Analysis of device PCE as a function of thermal annealing time at 85 °C for PCDTBT:PC_60_BM solar cells with conventional ITO/PEDOT:PSS/PCDTBT:PC_60_BM/Ca/Al architecture (black squares), inverted ITO/ZnO/PCDTBT:PC_60_BM/MoO_3_/Ag architecture (red circles) and the same inverted architecture subject to 165 minutes of 10 mW/cm^2^ white light exposure prior to thermal treatment (blue triangles). (**c**) PCE analysis as a function of thermal annealing time at 85 °C thermal stress for PCDTBT:PC_70_BM solar cells with conventional ITO/PEDOT:PSS/PCDTBT:PC_70_BM/Ca/Al architecture (black squares) and inverted ITO/ZnO/PCDTBT:PC_70_BM/MoO_3_/Ag architecture (red circles), depicted in Supplementary Fig. S1.

We now consider the substrate-dependence of the performance and stability of benchmark PCDTBT:PCBM OPV devices. Due to SiO_x_ being unsuitable for device fabrication, only PEDOT:PSS and ZnO substrates were investigated, and the thermal stability of completed devices systematically compared. (To ensure a reasonable comparison between *optimised* devices, Ca/Al and MoO_3_/Ag were selected as top interlayers/electrodes for PEDOT:PSS and ZnO based devices respectively due to their optimal workfunction. A systematic comparison of thermal ageing of pre- and post-annealed devices was performed to deconvolute the effects of top interlayer/electrode and morphological changes on OPV device thermal degradation, reported in Supplementary Fig. S7. When correlating film morphological behaviour to device thermal stability, control experiments were always performed to ensure that the device behaviour of pre-annealed devices matches that of post-annealed devices. Further, morphological studies of films and thermal stability studies of completed device always followed the same sample preparation and testing procedure). The evolution of current-voltage characteristics under 85 °C thermal stress for a typical PCDTBT:PC_60_BM device with a conventional ITO/PEDOT:PSS/PCDTBT:PC_60_BM/Ca/Al architecture is shown in Fig. 3(a). Due to the rapid initial degradation during the thermal burn-in process, the device suffers a larger PCE loss in the first 20 hours (25–30%) than the next 300 hour (≈10%) mainly due to losses in FF and Jsc associated, in part with an increase in device series resistance, resulting in an overall PCE loss of approximately 40% after 330 hour of 85 °C thermal stress, Fig. 3(b). In comparison, devices fabricated with an inverted ITO/ZnO/PCDTBT:PC_60_BM/MoO_3_/Ag architecture exhibit significantly improved thermal stability albeit with a small compromise (less than 10%) in initial PCE, Fig. 3(b). Specifically, inverted ZnO based devices experience a much smaller thermal burn-in in conjunction with reduced longer term degradation, with an overall PCE loss of only ≈10% after same period of 85 °C thermal stress. We have previously reported that light-induced PC_60_BM oligomerisation can also lead to an improved blend morphological– and hence device thermal stability^9^,^10^. Consistent with this observation, it is apparent that exposure to low level white light prior to thermal annealing further enhances the thermal stability of inverted ZnO based devices, resulting in an overall PCE loss of only ≈5% after 300 hour of 85 °C thermal stress with negligible thermal burn-in losses.

PCDTBT:PC_70_BM devices were also investigated to further demonstrate the generality of substrate dependent device thermal stability, with results shown in Fig. 3(c). Conventional PCDTBT:PC_70_BM devices based on PEDOT:PSS substrates exhibit a less severe thermal burn-in process than its PC_60_BM counterpart, with a PCE loss of 25–30% after 165 h of 85 °C thermal stress, consistent with ours and other previous reports^9^,^24^,^25^. By comparison, inverted PCDTBT:PC_70_BM devices based on ZnO substrates exhibit remarkable device thermal stability, with no observable thermal burn-in effect and negligible PCE loss after the same period of 85 °C thermal stress. This greatly improved thermal stability is striking and promising for the development of thermally stable OPV devices.

Our morphological and device results suggest a correlation between the degradation of device performance and the nucleation of PCBM crystallites at the nm-scale under modest thermal stress conditions. The formation of these nm-scale PCBM crystallites is likely related to the thermal “burn-in” in the early stages of device degradation, considering that these two processes occur within the same timescale (normally within the first 100–1000s of minutes). The physical origin of the substrate-dependent morphological evolution under thermal stress is not, however, obvious and may originate from multiple mechanisms. Component segregation to interfaces, both symmetric and asymmetrically, has been extensively documented in thin film, both supported and free standing, polymer mixtures and composites^26^. Notably, stratification is highly dependent upon different blend components, with for example, PCBM exhibiting enrichment adjacent to the PEDOT:PSS interface for P3HT:PCBM blends, while showing depletion for PCDTBT:PCBM blends^27^,^28^. The presence of an interface often results in preferential wetting of one component, which can in turn affect the local mobility profile, as well as the film stability to dewetting and can disrupt the isotropy of phase transformations, including crystallisation and demixing. We and others have reported that differences in surface energy of the substrate can result in different fullerene vertical segregation profiles (i.e. orthogonal to the film surface) and, in turn, different crystallisation behaviour^9^,^29^,^30^,^31^,^32^. The surface energy of the substrates (Supplementary Fig. S3) appears to provide a favourable correlation with the morphological observations with γ(SiO_x_) > γ(ZnO) > γ(PEDOT:PSS), yielding progressively fewer micro-crystals and more nano-crystals, within the temperature range investigated. ZnO appears to hinder both crystallisation mechanisms within this temperature range.

**Figure 4:**
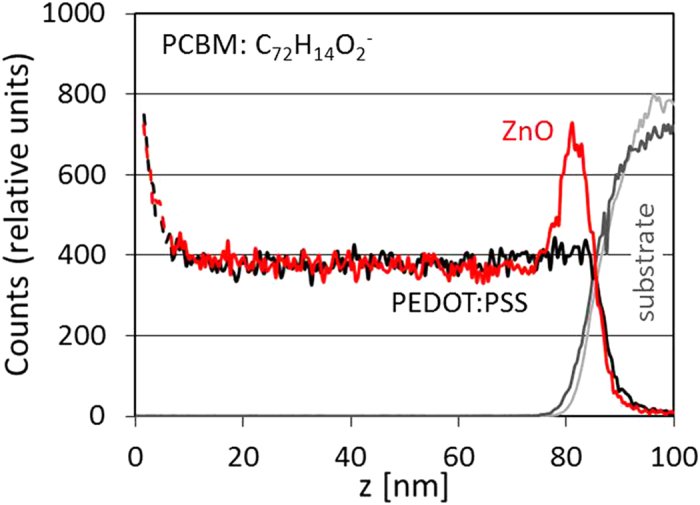
TOF-SIMS results of the PC_60_BM trace (C_72_H_14_O_2_^−^) of PCDTBT:PC_60_BM films cast onto PEDOT:PSS (black) and ZnO (red) substrates. The substrate interfaces are identified by the C_8_H_7_SO_3_^−^ and ZnO^−^ traces for the PEDOT:PSS (dark grey) and ZnO (light grey) substrates, in relative units. PC_60_BM enrichment at the ZnO substrate is observed, with respect to PEDOT:PSS.

We therefore examined the compositional segregation of PCDTBT:PCBM on a variety of substrates and annealing conditions using Time-of-Flight Secondary Ion Mass Spectrometry (TOF-SIMS) and neutron reflectivity. Figure 4 shows the TOF-SIMS PC_60_BM trace obtained for as-cast blend films on PEDOT:PSS and ZnO substrates. Films deposited on ZnO exhibit a distinct PC_60_BM enrichment at the substrate interface; in contrast this enrichment is not observed for blends deposited on PEDOT:PSS. Thermal annealing at 80 °C does not alter the profiles significantly (Supplementary Fig. S8). One can thus expect that at modest thermally annealing temperatures, below the polymer T_g_ (106 °C for PCDTBT), PCBM only has limited mobility and only nanoscale crystallisation is possible. The higher PCBM segregation at the ZnO substrate interface further hinders the formation of nm-sized PCBM crystallites due to its reduced mobility for PCBM diffusion/crystallisation. At sufficiently high temperatures, above the blend T_g_, PCBM evidently has sufficiently high mobility to result in macroscopic crystallisation, as shown in Fig. 1(a). Films supported on PEDOT:PSS undergo nano-sized crystallisation at lower thermal stresses (Fig. 2e,g), presumably due to the competitive nature of bimodal PCBM crystallisation^16^, yielding fewer micron-sized crystallites at higher thermal stress (Fig. 1a). The top surface enrichment and nanoscale crystallisation of PCBM on PEDOT:PSS substrates also suggest that nano-crystals likely form away from the substrate, within the bulk of the film. The neutron reflectivity scattering length density depth profile of PCDTBT:PCBM on SiO_x_ (in comparison with PEDOT:PSS, see Supplementary Fig. S9) suggests that a surface energy mediated higher local PCBM concentration adjacent to the substrate, coupled with a higher mobility of PCBM within the polymer matrix, may facilitate micron-scale crystallisation. Our results also suggest that the micron-scale and nanoscale crystallisation of PCBM are largely mutually exclusive events (i.e. mostly micron-scale crystallisation accompanies few nano-sized crystallites, or vice versa), caused by the substrate dependent surface enrichment of PCBM at either top or bottom interface.

An additional factor contributing to the different PCBM crystallisation behaviour on PEDOT:PSS and ZnO substrates could be their large difference in surface roughness. As shown in Fig. 2(a,b), ZnO substrates possess a much larger roughness than PEDOT:PSS, with a length scale comparable to that of nm-scale PCBM crystallites. These ZnO asperities could potentially disrupt the formation of critical PCBM nuclei, and thus also frustrate nucleation. Evidently, establishing a universal correlation between substrate roughness, surface energy, fullerene segregation and blend thermal stability appears complex, partially due to challenges in decoupling these parameters while retaining functional devices with comparable performance, and also due to the bimodal nature of thermally activated nucleation and growth of PCBM crystallites. Other effects may also have to be considered: for example, we have recently found that polymer polydispersity (PDI) can have a large impact on the formation of nm-scale PCBM crystallites formation.

It should also be noted that Sachs-Quintana *et al.* have reported the formation of a thin, polymer rich layer at temperatures above the polymer glass transition temperature, which acts as an electron barrier and may also contribute to the thermally induced degradation of OPV devices^18^. It is apparent that thermally induced degradation (especially the thermally induced “burn-in” losses) of OPV devices has complex origins and is dependent upon several parameters, and therefore further studies (for example, examination of a broader range of device interlayers) are desirable. Nevertheless, our correlations for subsets of well-defined experimental conditions appear robust and provide insight into the relation between morphological and device stability in polymer/fullerene blends, and in particular the role of PCBM crystallite nucleation in determining this stability.

In conclusion, we demonstrate that the morphological stability of organic solar cells under thermal stress is strongly dependent upon the bottom substrate interface on which the active layer is deposited. PCBM crystallisation at both the micron- and nano-scale appears to correlate with differences in surface energy (and potentially surface roughness), with ZnO exhibiting minimal crystallisation for both mechanisms within the temperature range studied. With this approach, we demonstrate that inverted OPV device structures employing a ZnO electron collection layer can exhibit, in addition to previously reported improved resistance to humidity induced degradation, remarkably improved morphological and device stability under 85 °C thermal stress. For PCDTBT:PC_60_BM photoactive layers, this thermal stability is further improved by modest light pre-exposure. For inverted PCDTBT:PC_70_BM devices, no measurable (<2%) degradation of performance was observed over 180 hour of 85 °C thermal stress. This study thus demonstrates that selection of suitable device electrodes can substantially enhance the morphological stability of the photoactive layer and thus the thermal stability of organic solar cells.

## Methods

### Materials

PCDTBT was supplied by 1-Materials and purified at Imperial with a molecular weight (M_w_) of 21.6 kgmol-1 and polydispersity (PDI) of 5.5. The glass transition temperature T_g_ of neat PCDTBT was measured as 106 ± 1 °C using different scanning calorimetry (DSC) at a rate of 10 °C/min. PC_60_BM was supplied by Nano-C Inc. and PC_70_BM was supplied by Solene BV.

### Sample preparation

All substrate types were prepared on SiO_x_ wafers (cleaned with N_2_ before use). For ZnO substrates preparation, ZnO precursor solution was prepared by codissolving 219.5 mg of Zinc acetate dehydrate, 2 ml of 2-methoxyethanol and 60.4 μl of ethanolamine and stirring overnight at room temperature. Crystalline ZnO films were formed by spin coating the precursor solution onto SiO_x_ wafers followed by thermal annealing ay 150 °C for 20 minutes in ambient environment. PEDOT:PSS substrates were prepared by spin coating PEDOT:PSS solution onto SiO_x_ wafers followed by thermal annealing at 150 °C for 20 minutes. Both the PEDOT:PSS and ZnO substrates have an interlayer thickness of ~ 35 nm, as measured with a Dektak 6 M profilometer.

### Morphological studies

PCDTBT and PC_60_BM (1:2 weight ratio) was codissolved in chlorobenzene (25 mg/ml total concentration) and stirred overnight at 55 °C inside a N_2_ glovebox. The blend films were prepared by spin coating the blend solution after filtration through a 0.2 μm PTFE filter, resulting in film thickness of 85–100 nm for all substrate types. All blend films were annealed inside a N_2_ filled glovebox with annealing temperatures measured and calibrated with a surface thermocouple (Kane-May KM330). Optical images of the blend morphologies were obtained by reflection optical microscope (Olympus BX 41M), equipped with an XY stage and CCD camera (AVT Marlin). Surface topography roughness of the blend films were acquired using atomic force microscopy (Innova Bruker AXS) in tapping mode, using super sharp TESP-SS tips. Experimental procedures are illustrated in Supplementary Fig. S2.

### OPV device fabrication and thermal stability studies

ITO glass substrates were cleaned with detergent solution, acetone and isopropanol successively, followed by oxygen plasma treatment at 100 W for 7 minutes. Both the substrate interlayers (PEDOT:PSS and ZnO) and blend active layers (PCDTBT:PC_60_BM and PCDTBT:PC_70_BM) were prepared following the same deposition method described above. Conventional PEDOT:PSS devices were completed by thermal evaporation of 25 nm of calcium and 100 nm of aluminum, while inverted ZnO devices were completed by thermal evaporation of 10 nm of MoO_3_ and 100 nm of silver through a 6-pixel mask with a well-defined pixel area of 0.045 cm^2^ (configuration illustrated in Supplementary Fig. S1). Light processing for inverted PCDTBT:PC_60_BM devices were performed using a desktop fluorescence lamp with a light intensity of 10 mW/cm^2^ and duration of 165 minutes. Device thermal stability studies were performed in a N_2_ glovebox following the same method as the morphological studies described above. Current density-voltage (J-V) characteristics were recorded using a Xenon lamp at AM1.5 solar illumination (Oriel Instruments) calibrated to a silicon reference cell with a Keithley 2400 SMU.

### Time of flight secondary ion mass spectrometry studies

Depth profiling measurements were carried out using an IOTOF ToF-SIMS 5 dual beam instrument. The analytical primary ion beam was a 25 keV Bi_3_^+^ with a current of 0.25 pA at 150 μs in high current bunched mode for high mass resolution. The secondary sputtering ion beam was an argon cluster of 1000 atoms, Ar_1000_, at 2.5 keV and a current of 1nA. The sample area sputtered was 300 μm^2^, and the Bi_3_^+^ analytical area was 200 μm^2^. Negative secondary ions were collected.

### Surface energy and contact angle measurements

Surface energy for the various substrates was calculated by the Owens, Wendt, Rabel and Kaeble method, based on static contact angle measurements (Easydrop, Kruss GmbH) with 3 μl of deionised water, methylene iodide (Sigma Aldrich) and glycerol (Sigma Aldrich).

## Additional Information

**How to cite this article**: Li, Z. *et al.* Toward Improved Lifetimes of Organic Solar Cells under Thermal Stress: Substrate-Dependent Morphological Stability of PCDTBT:PCBM Films and Devices. *Sci. Rep.* **5**, 15149; doi: 10.1038/srep15149 (2015).

## Supplementary Material

Supplementary Information
