# Supplementary material for: Toward Improved Lifetimes of Organic Solar Cells under Thermal Stress: Substrate-Dependent Morphological Stability of PCDTBT:PCBM Films and Devices
Source: Sci Rep. 2015 Oct 15;5:15149. doi: 10.1038/srep15149 (PMC4606801; doi:10.1038/srep15149)
Supplement: Supplementary Information [file srep15149-s1.pdf]

Supplementary Information

# Toward Improved Lifetimes of Organic Solar Cells under Thermal Stress: Substrate-Dependent Morphological Stability of PCDTBT:PCBM Films and Devices

**Zhe Li+[1,4], Kar Ho Chiu+[1,2], Raja Shahid Ashraf[1], Sarah Fearn[3], Rajeev Dattani[2], Him Cheng Wong[2,5], Ching-Hong Tan[1,4], Jiaying Wu[1], João T. Cabral[2]* and James R. Durrant[1,4]***

[1] Department of Chemistry, Centre for Plastic Electronics, Imperial College London, London SW7 2AZ.
[2] Department of Chemical Engineering, Centre for Plastic Electronics, Imperial College London, London SW7 2AZ.
[3] Department of Materials, Centre for Plastic Electronics, Imperial College London, London SW7 2AZ.
[4] SPECIFIC, College of Engineering, Swansea University, Baglan Bay Innovation, Knowledge Centre, Central Avenue, Baglan SA12 7AX.
[5] Engineering Product Development, Singapore University of Technology and Design, 8 Somapah Road, 487372 Singapore.
* j.cabral@imperial.ac.uk,j.durrant@imperial.ac.uk.
+these authors contributed equally to this work

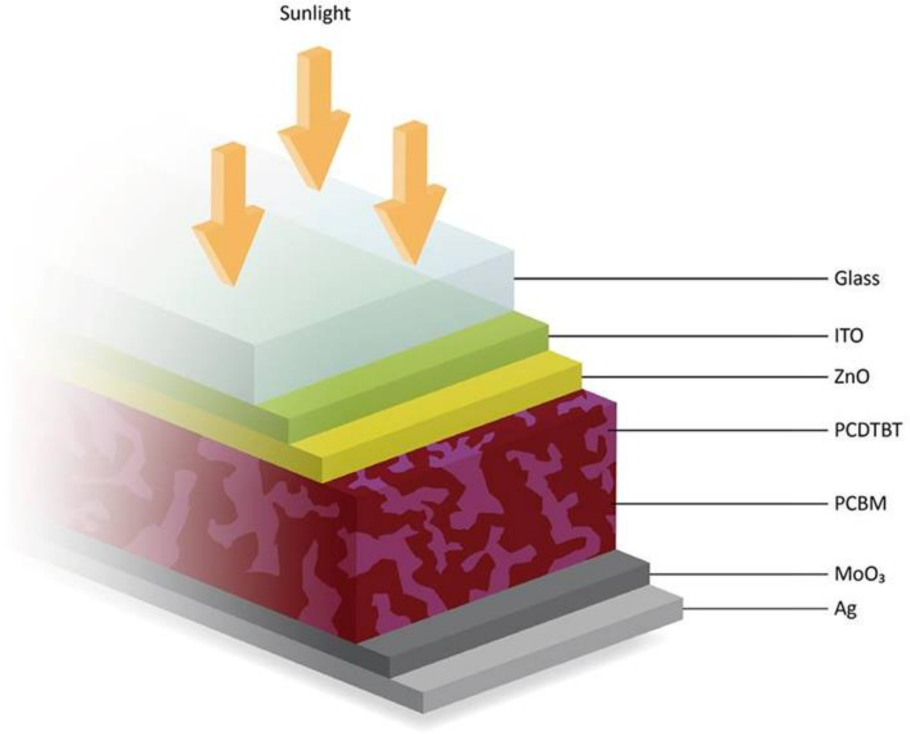


**Figure S1.** Organic solar cell configuration of inverted ZnO devices.

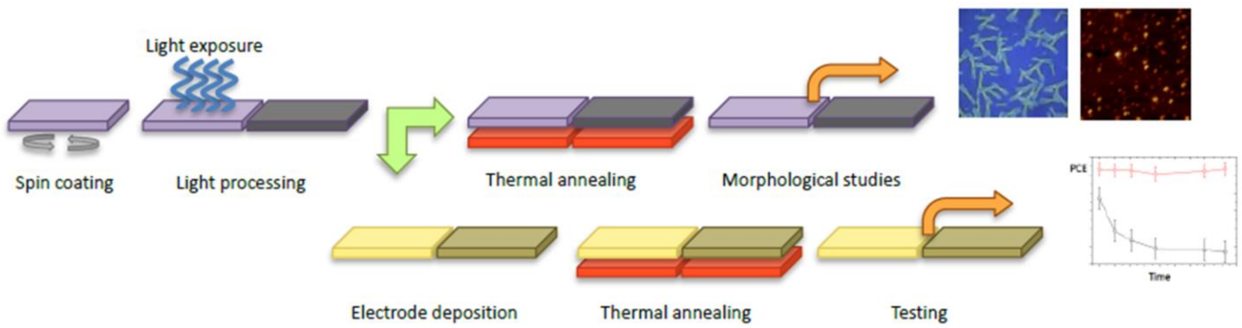


**Figure S2.** Protocol followed in this work for morphological quantification (top row) and device performance evaluation (bottom row).

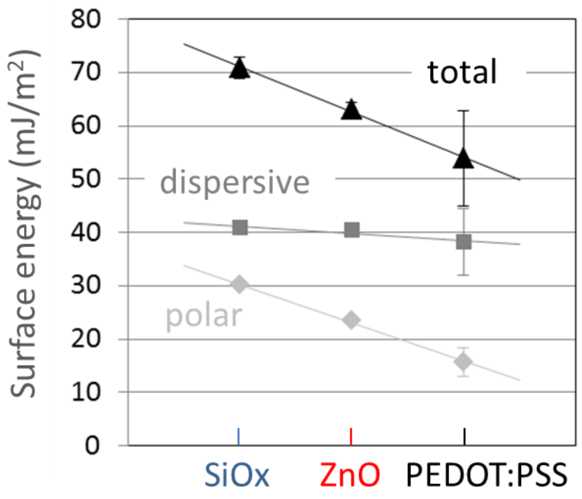


**Figure S3.** Surface energy of substrates SiOx, ZnO, PEDOT:PSS, including polar and dispersive contributions. The larger uncertainty for PEDOT:PSS likely arises from environmental conditions/ hygroscopy.

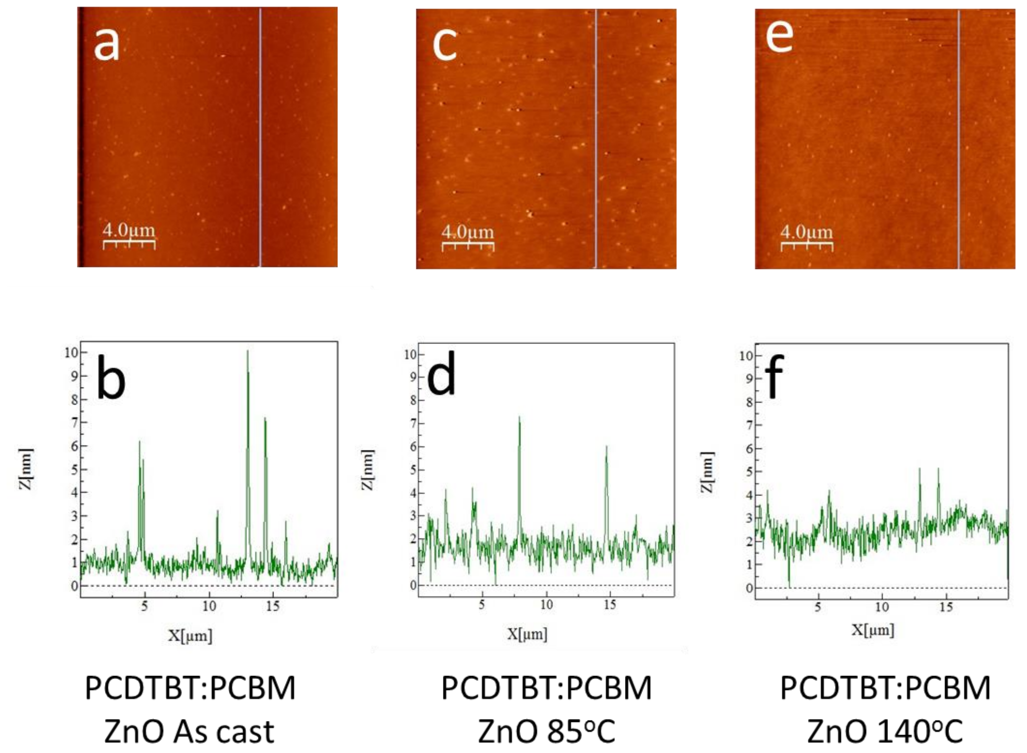


**Figure S4.** Line scans of AFM images of PCDTBT:$PC_{60}BM$ (1:2) blend films without thermal annealing (a,b), blend films thermally annealed at 85$^{o}$C (c,d) and 140$^{o}$C (e,f) on ZnO substrates. The RMS roughness was measured as 2.03nm, 1.06nm and 0.74nm for as cast blend films, blend films thermally annealed at 85$^{o}$C, and blend films thermally annealed at 140$^{o}$C respectively.

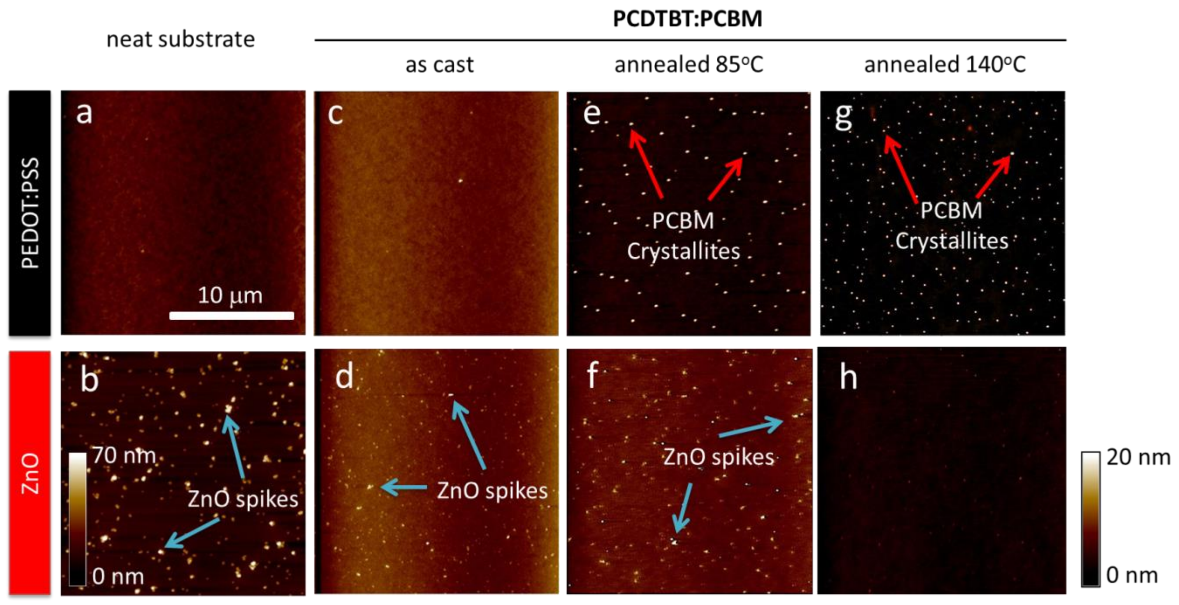


**Figure S5.** AFM images with arrows distinguishing nanoscale ZnO features and $PC_{60}BM$ crystallites.

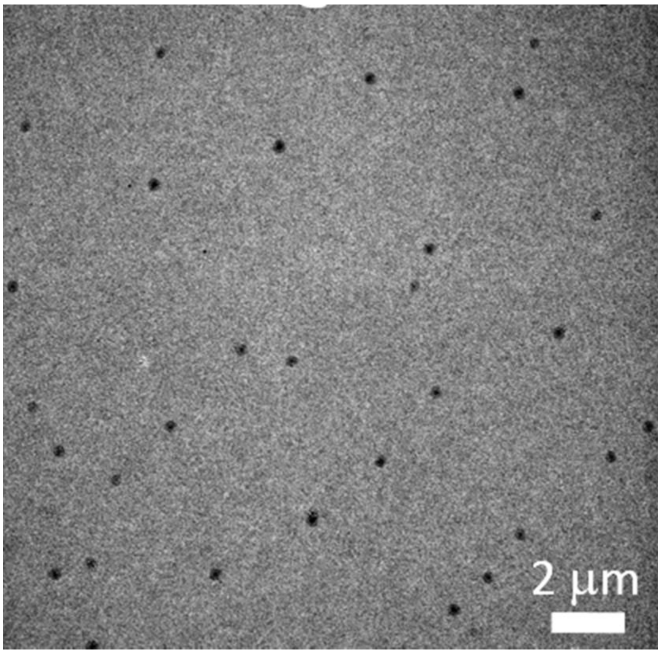


**Figure S6.** TEM image of thermally annealed PCDTBT:$PC_{60}BM$ blend films on PEDOT:PSS; the greater contrast of the image corresponds to the large mass and electron density expected for PCBM (approx. 1.5 $g/cm^3$) compared to the polymer.

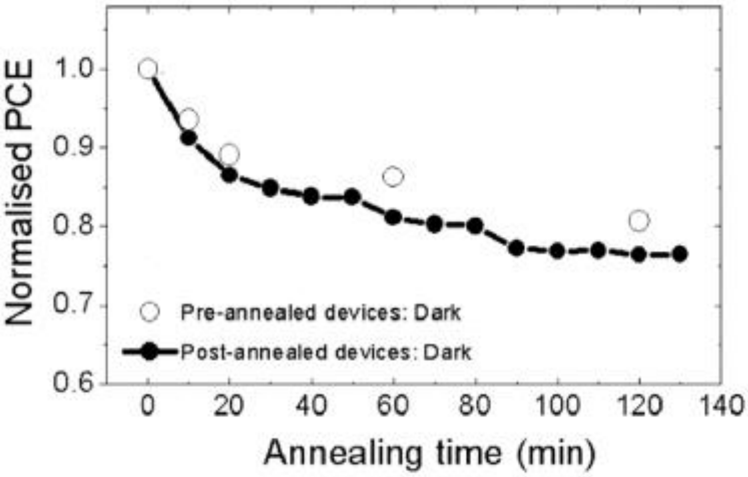


**Figure S7.** Systematic comparison of thermal ageing of pre- and post-annealed devices to deconvolute the effects of top interface layer/electrode and morphological changes on thermal degradation of OPV devices. This figure shows that both pre- and post-annealed devices show similar thermal stability behavior under thermal stress, inferring that the influence of top electrode on device thermal stability is insignificant, at least for the high $T_g$ PCDTBT based blend system studied herein.

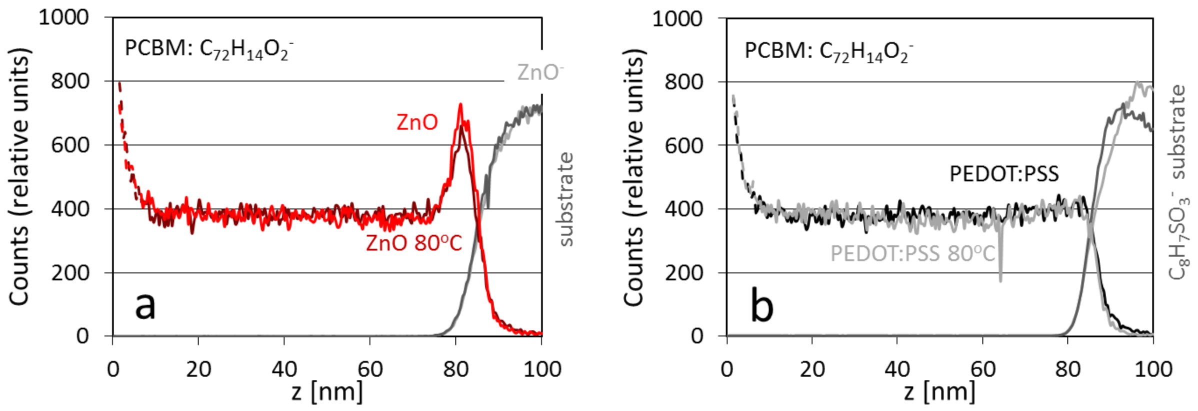


**Figure S8.** TOF-SIMS results for PCDTBT:PCBM (1:2) films on (a) ZnO and (b) PEDOT:PSS substrates upon thermal annealing at 80°C for 1h. The PCBM content is traced by the $C_{72}H_{14}O_2^-$ signal along the depth [z] of the film. The substrates are located by the ZnO- , SiO2 and $C_8H_7SO_3$- signals for ZnO, SiOx and PEDOT:PSS respectively, and are shown in relative units, commensurate with the $C_{72}H_{14}O_2^-$ trace. No significant changes of PCBM segregation profile as observed on either substrate: (a) PCBM remains segregated to the ZnO interface is observed, and marginally to the PEDOT:PSS interface.

**Neutron reflectivity** measurements were performed on the D17 reflectometer at Institut Laue-Langevin (ILL), Grenoble, in time-of-flight (TOF) mode using a wavelength range of 2 Å to 20 Å ($\Delta\lambda/\lambda$ ~ 4%) and two incident angles of incidence (0.6 and 2.4 °), obtaining a wavenumber qz window of $0.004 < qz < 0.22$ Å$^{-1}$. Data analyzed using Parratt32 (v 1.6, HMI Berlin).

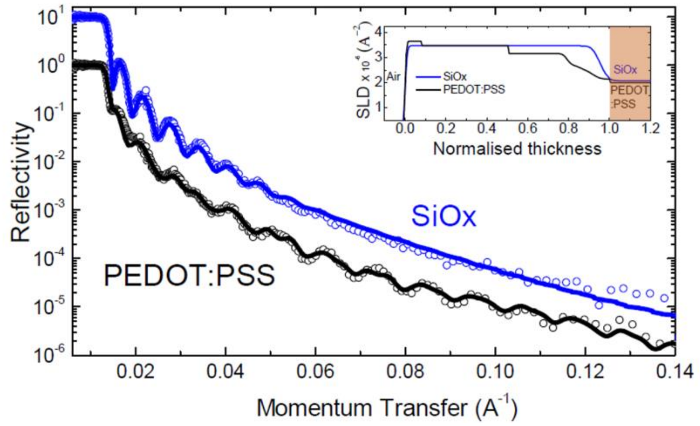


**Figure S9.** Neutron reflectivity profiles of PCDTBT:$PC_{60}BM$ films spun cast on SiOx and PEDOT:PSS substrates. Inset shows the corresponding scattering length density (SLD) profiles showing a broadly uniform PCBM profile in $SiO_x$ and an asymmetrically segregated profile (towards the air interface) on PEDOT:PSS.
